# Physicochemical, Phytochemical, and Toxicological Assessment of *Agrimonia pilosa*, *Calendula arvensis*, and *Polygonum hydropiper* Tinctures with Hypoglycemic Potential

**DOI:** 10.3390/molecules31132316

**Published:** 2026-07-01

**Authors:** Roxana Kostici, Adina Maria Kamal, Diana-Maria Trasca, Carmen Vladulescu, Renata Maria Varut, Pluta Ion Dorin, Daniela Cîrțînă, Maria Stoica, Gabriela Pura, Romeo Popa, Mihaela Popescu, Pirscoveanu Denisa Floriana Vasilica

**Affiliations:** 1 Department of Toxicology, Faculty of Pharmacy, University of Medicine and Pharmacy of Craiova, 200349 Craiova, Romania; roxana.kostici@umfcv.ro; 2 Department of Internal Medicine, Faculty of Medicine, University of Medicine and Pharmacy of Craiova, 200349 Craiova, Romaniadiana.trasca@umfcv.ro (D.-M.T.); 3 Department of Biology and Environmental Engineering, Faculty of Horticulture, University of Craiova, 200585 Craiova, Romania; 4 Research Methodology Department, Faculty of Pharmacy, University of Medicine and Pharmacy of Craiova, 200349 Craiova, Romania; 5 Faculty of Medical and Behavioral Sciences, Constantin Brâncuși University of Târgu Jiu, 210185 Târgu Jiu, Romania; dorin.pluta@e-ucb.ro; 6 Department of Intensive Care and Anesthesia, Emergency County Hospital, Faculty of Medicine, University of Medicine and Pharmacy of Craiova, 200349 Craiova, Romania; 7 Department of Medical Devices and Pharmaceutical Practice, Iuliu Hațieganu University of Medicine and Pharmacy, 400012 Cluj-Napoca, Romania; 8 Department of Pharmacology, Faculty of Medicine, University of Medicine and Pharmacy of Craiova, 200349 Craiova, Romania; 9 Department of Endocrinology, Faculty of Medicine, University of Medicine and Pharmacy of Craiova, 200349 Craiova, Romania; 10 Department of Neurology, Faculty of Medicine, University of Medicine and Pharmacy of Craiova, 200349 Craiova, Romania

**Keywords:** medicinal plants, hypoglycemic activity, polyphenols, antioxidant activity, toxicity assessment, diabetes mellitus:

## Abstract

Diabetes mellitus represents a major global health burden, necessitating the development of safer and more effective therapeutic alternatives. Medicinal plants have gained increasing attention due to their bioactive compounds with potential hypoglycemic and antioxidant effects. The present study aimed to investigate the physicochemical characteristics, phytochemical composition, antioxidant capacity, and toxicological profile of hydroalcoholic tinctures obtained from *Agrimonia pilosa* Ledeb., *Calendula arvensis* L., and *Polygonum hydropiper* L. The tinctures were prepared by simple percolation using 70% ethanol and evaluated according to pharmacopoeial standards, including organoleptic properties, relative density, refractive index, alcohol content, and purity parameters. Phytochemical analysis was performed using thin-layer chromatography and spectrophotometric methods, highlighting the presence of flavonoids and polyphenolcarboxylic acids, with several bands showing chromatographic and spectral similarities to chlorogenic and caffeic acid standards. Antioxidant activity was assessed through total polyphenol and flavonoid content, with *Polygonum hydropiper* exhibiting the highest values. The hypoglycemic effect was evaluated using the oral glucose tolerance test in normoglycemic mice, demonstrating significant reductions in blood glucose levels, particularly for *Agrimonia pilosa* at higher doses. Acute toxicity studies indicated a low toxicity profile, with no mortality observed even at high doses (up to 9 g/kg body weight), corresponding to GHS category 5. However, subacute toxicity assessment revealed species-dependent effects, ranging from minimal hepatic changes for *Calendula arvensis* to moderate hepatotoxicity for *Polygonum hydropiper* and more pronounced hepatic, renal, and pancreatic alterations for *Agrimonia pilosa*. These findings suggest that the investigated tinctures possess significant hypoglycemic and antioxidant potential, with generally favorable safety profiles following acute administration. Nevertheless, prolonged use may induce organ-specific toxicity, highlighting the need for further pharmacological and clinical investigations to establish their therapeutic applicability and safety in diabetes management.

## 1. Introduction

Diabetes mellitus represents a major global health concern affecting individuals across all social and economic backgrounds [1,2,3,4,5]. Its global prevalence has increased to epidemic proportions, driven by population growth, aging, urbanization, obesity, and sedentary lifestyles [6]. Over time, substantial progress has been made in the understanding, diagnosis, and classification of diabetes mellitus, as reflected in successive reports and updates issued by the World Health Organization and the American Diabetes Association [7,8,9,10,11]. Diabetes is generally defined as a group of metabolic disorders characterized by chronic hyperglycemia resulting from impaired insulin secretion, reduced insulin action, or both, and is associated with disturbances in carbohydrate, protein, and lipid metabolism [10,12,13,14,15]. Persistent hyperglycemia is linked to long-term damage, dysfunction, and failure of several organs, particularly the eyes, kidneys, nerves, heart, and blood vessels [16,17]. Although historical classifications distinguished between insulin-dependent, non-insulin-dependent, gestational, and secondary diabetes [18,19], current classifications recognize type 1 diabetes, type 2 diabetes, gestational diabetes, and other specific types with diverse etiologies [20,21,22,23,24].

In parallel with conventional medicine, medicinal plants have long been used as traditional remedies for diabetes management in different parts of the world [25]. Their use is supported by their availability, complex phytochemical composition, and potential to provide bioactive compounds with complementary pharmacological effects [26]. Approximately 350 medicinal plants are used in traditional diabetes management in various pharmaceutical forms, including extracts, decoctions, herbal mixtures, and powders prepared from different plant organs [27]. The antidiabetic potential of phytochemicals has been associated with several mechanisms reported in the literature, including reduction in intestinal glucose absorption, inhibition of hepatic glucose production, enhancement of peripheral glucose uptake, stimulation of insulin secretion, modulation of insulin-related enzymes, and protection or regeneration of pancreatic β-cells [28]. In addition to direct hypoglycemic effects, antioxidant, anti-inflammatory, antiapoptotic, and antiglycation activities may contribute to the prevention of diabetes-related complications [29,30]. Although ethnopharmacology recognizes more than 1200 plant species with hypoglycemic activity, research into plant-derived antidiabetic agents remains relevant due to their potential to provide alternative or complementary therapeutic options [31,32]. Many of these plants contain biologically active compounds such as glycosides, alkaloids, terpenoids, flavonoids, and carotenoids, which may contribute to their pharmacological effects [33].

Among the medicinal plants investigated in the present study, *Agrimonia pilosa* Ledeb., *Calendula arvensis* L., and *Polygonum hydropiper* L. were selected based on their traditional medicinal use, availability, and reported richness in flavonoids and phenolic compounds. These phytochemical classes are commonly associated with antioxidant activity and may contribute to glucose-lowering effects. Therefore, the three species were comparatively evaluated as hydroalcoholic tinctures with potential relevance for complementary diabetes management.

The diverse chemical composition of the studied plant species, together with their documented medicinal uses and the potential for cumulative toxicity upon prolonged administration, provided the basis for the preparation and physicochemical characterization of tinctures with hypoglycemic potential. The tinctures were obtained by simple percolation and evaluated according to the Romanian Pharmacopoeia, including organoleptic characteristics, relative density, refractive index, alcohol content, residue on evaporation, and purity parameters. Qualitative and quantitative analyses of flavonoids and phenolic acids were performed using thin-layer chromatography and spectrophotometric methods, while antioxidant capacity was assessed by determining total phenolic and flavonoid content.

The novelty of the present study lies in the comparative evaluation of hydroalcoholic tinctures prepared from *Agrimonia pilosa*, *Calendula arvensis*, and *Polygonum hydropiper* under pharmacopoeial conditions. Unlike previous studies focusing mainly on isolated extracts or individual biological effects, this work integrates physicochemical characterization, TLC phytochemical profiling, spectrophotometric quantification of phenylpropanoid compounds, antioxidant assessment, oral glucose tolerance testing, and acute/subacute toxicity evaluation in the same experimental framework.

## 2. Results

The main physicochemical characteristics of the tinctures obtained from plant materials with hypoglycemic properties are summarized in Table 1, Table 2, Table 3 and Table 4.

The analysis of the experimental results revealed that all tinctures investigated contained both flavonoids and polyphenolcarboxylic acids, with certain components being identified based on the presence of characteristic chromatographic bands (Figure 1, Figure 2, Figure 3, Figure 4, Figure 5, Figure 6, Figure 7, Figure 8 and Figure 9).

In the case of flavonoid analysis, although flavonoids were detected in all analyzed tinctures, the lack of appropriate reference standards did not allow their precise identification.

Regarding the analysis of polyphenolcarboxylic acids, the chromatographic and UV spectral data indicated different degrees of similarity with the reference standards. In the APH tincture, a major band was observed at Rf 0.27, close to the migration zone of chlorogenic acid; however, the corresponding UV spectrum did not fully match the chlorogenic acid standard. Therefore, this band was not considered definitively identified as chlorogenic acid, but rather tentatively assigned to a chlorogenic acid-related hydroxycinnamic derivative or to a co-migrating phenolic compound. In contrast, the bands observed in CAH and PHH showed better agreement with the chlorogenic acid standard based on Rf values and UV spectral similarity and were therefore putatively assigned to chlorogenic acid. Caffeic acid was also putatively detected in PHH based on chromatographic behavior and UV spectral comparison. Other polyphenolcarboxylic compounds were suggested by distinct chromatographic bands; however, their precise identification was not possible using TLC/UV data alone. Furthermore, Table 5 summarizes the results concerning the in vitro antioxidant activity of the analyzed tinctures, previously diluted in a 1:5 ratio with distilled water, as evaluated based on their total polyphenol and total flavonoid content.

The total polyphenol content of the analyzed tinctures, expressed as mg/L gallic acid equivalents (GAE), was found to be directly correlated with their in vitro antioxidant activity. Among the investigated samples, the highest value was recorded for the PHH tincture (328.35 ± 6.08), followed by APH (280.58 ± 5.57), while CAH exhibited a significantly lower polyphenol content (97.05 ± 1.94).

A similar trend was observed for the total flavonoid content, expressed as mg/L quercetin equivalents (QE), with PHH showing the highest concentration (151.65 ± 3.03), followed by APH (138.29 ± 2.76) and CAH (113.52 ± 2.27). These findings further support the relationship between the concentration of phenolic compounds and the antioxidant potential of the analyzed tinctures.

The results of the chemical analyses highlight notable differences in the phytochemical profiles of the investigated samples and emphasize the relevance of polyphenols and flavonoids as key contributors to antioxidant activity. These data support the need for further pharmacodynamic and biochemical investigations aimed at confirming the therapeutic value of the analyzed tinctures, particularly in relation to their potential hypoglycemic and antioxidant effects.

Moreover, the relatively limited number of studies focusing on hydroalcoholic extracts derived from *Agrimonia pilosa* Ledeb., *Calendula arvensis* L., and *Polygonum hydropiper* L. further underlines the importance of continued research in this field.

### 2.1. Acute Toxicity Assessment in Mice for the Investigated Plant Tinctures

In the first experimental group, mice received, by oral gavage, a tincture prepared from *Calendula arvensis* L. at doses of 1, 2, 3, 4, and 5 g/kg body weight. Within 5–10 min after administration, animals treated with doses of 3, 4, and 5 g/kg exhibited signs of somnolence and tachycardia. One hour after administration, all mice, regardless of dose, were observed to be asleep. After approximately two hours, the animals regained consciousness and resumed normal feeding behavior. At 24 h post-administration, no abnormal behavioral changes were observed.

In the third experimental group, mice were administered tincture of *Agrimonia pilosa* Ledeb. at doses of 1, 2, 3, 4, and 5 g/kg body weight. Initially, somnolence was observed only in animals receiving the highest dose (5 g/kg). However, at 45 min post-administration, all animals were asleep. Full recovery occurred approximately 70 min after administration, and at 24 h all animals exhibited normal behavior without any observable adverse effects.

In the group receiving tincture of *Polygonum hydropiper* L. at equivalent dose levels, a similar pattern was observed. Somnolence and tachycardia appeared shortly after administration at higher doses, followed by a transient sleep phase affecting all animals. Recovery occurred within approximately 1–2 h, and no behavioral or physiological abnormalities were noted after 24 h.

Clinical monitoring continued for up to 72 h, during which no animals exhibited any of the adverse reactions described in the monitored parameter list, including neurological, respiratory, cardiovascular, or gastrointestinal disturbances.

Following the initial acute toxicity assessment, the majority of animals were retained for further evaluation. From each group of ten animals, eight were selected, excluding those that had previously received the highest dose of 5 g/kg body weight. These animals were then subdivided into groups of two and administered higher doses of the respective tinctures (6, 7, 8, and 9 g/kg body weight) by oral gavage.

On the fourth day following the initial experiment, the animals were subjected to overnight fasting starting at 20:00. On the following day, between 09:00 and 10:00, body weight measurements were recorded, and the procedure was repeated after 24 h. Blood glucose levels were determined prior to tincture administration and again at 24 h post-administration (Table 6).

The obtained results indicated that even at higher doses, the tested tinctures did not induce acute toxic effects or mortality, suggesting a relatively low acute toxicity profile.

Immediately after administration, all animals exhibited signs of somnolence. Within 30 min, all mice entered a sleep state. After approximately two hours, the animals gradually regained consciousness, presenting mild motor incoordination during the recovery phase. At 24 h post-administration, all animals displayed normal behavior, including regular feeding and normal locomotor activity.

A slight decrease in body weight, ranging between 1–2 g, was observed in almost all animals after 24 h. Blood glucose levels showed an increase at 24 h post-administration; however, these values remained within physiological limits.

Shortly after administration of the tincture, all mice exhibited pronounced sedation and fell asleep. At two hours post-administration, the animals remained somnolent. However, after 24 h, all mice displayed normal behavior, with no evident clinical abnormalities. A decrease in body weight of approximately 1.5–2 g was observed in all animals at 24 h. In five mice, blood glucose levels measured 24 h after administration were lower than baseline values, particularly at the dose of 8 g/kg body weight, where a marked reduction of up to 60% was recorded in one subject.

All mice initially exhibited signs of agitation, followed by the onset of somnolence. At 24 h post-administration, all animals displayed normal behavior, with no observable clinical abnormalities. A decrease in body weight of approximately 1.5–2 g was recorded in all subjects after 24 h. In six out of eight mice, blood glucose levels were slightly increased at 24 h compared to baseline values, with variations of up to 14%. However, at the dose of 8 g/kg body weight, a reduction in glycemia of approximately 15.7% was observed after 24 h.

### 2.2. Subacute Toxicity Assessment in Mice for the Investigated Plant Tinctures

The evaluation of water intake was performed for all experimental groups during the first week of the study (days 1–7), as part of the subacute toxicity assessment protocol. Mean water consumption during the first week was relatively similar across all experimental groups and the control group. The recorded values ranged between 22.86 ± 4.88 mL/week in group 2 and 25.00 ± 11.18 mL/week in group 3, while group 1 showed a mean intake of 24.29 ± 11.34 mL/week. The control group exhibited a slightly higher mean water consumption of 27.96 ± 9.06 mL/week (Table 7). Overall, no marked differences in water intake were observed between the treated and control groups during this period. The statistical comparison of weekly mean water consumption did not reveal significant differences between week 1 and week 2 across any experimental group (Wilcoxon signed-rank test, *p* > 0.05). These findings suggest that the investigated tinctures did not induce alterations in hydration behavior or general physiological status. Statistical analysis of food consumption across experimental groups during subacute toxicity assessment.

Group 3 (*Agrimonia pilosa*) showed a statistically significant difference in food consumption compared with the control group during the first week, with a lower mean weekly intake (17.57 g vs. 21.07 g; *p* < 0.05). In contrast, groups 1 and 2 did not show statistically significant differences compared with the control group (*p* > 0.05), corresponding to *Calendula arvensis* and *Polygonum hydropiper* (Table 8). While no significant differences in food consumption were observed for groups 1 and 2 compared with the control (*p* > 0.05), group 3 showed a statistically significant reduction (*p* = 0.011). This finding may indicate a potential effect of the corresponding extract on appetite regulation or metabolic activity.

During the second week, mean food consumption ranged between 21.00 ± 1.29 g in the *Agrimonia pilosa* group and 23.50 ± 1.19 g in the *Polygonum hydropiper* group, compared with 23.21 ± 2.20 g in the control group.

A statistically significant reduction in food consumption was observed in the *Agrimonia pilosa* group compared with the control during the second week (*p* = 0.038). In contrast, no significant differences were detected for the *Calendula arvensis* and *Polygonum hydropiper* groups (*p* > 0.05). The analysis of food consumption over time revealed a statistically significant increase in the *Polygonum hydropiper* group during the second week compared with the first week (*p* = 0.043). In contrast, no significant changes were observed in the *Calendula arvensis*, *Agrimonia pilosa*, or control groups (*p* > 0.05), suggesting a group-specific effect.

No statistically significant differences in body weight were observed between the *Calendula arvensis*-treated group and the control group throughout the 14-day experimental period (*p* > 0.05).

Similarly, the *Polygonum hydropiper* group did not show significant differences compared with the control, with the exception of a single time point (day 3), where a transient difference was detected (*p* < 0.05).

In the case of *Agrimonia pilosa*, no consistent changes in body weight were observed; however, isolated statistically significant decreases were recorded on days 3, 7, 10, and 11 (*p* < 0.05). These variations were not sustained over time, suggesting the absence of a clear treatment-related effect on body weight (Table 9).

**Figure 10:**
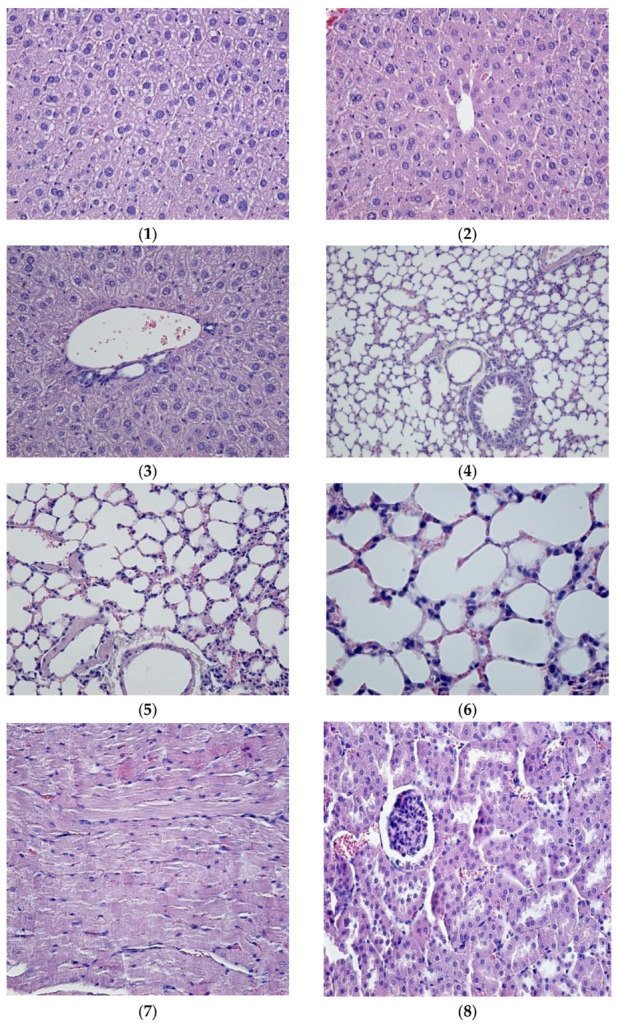

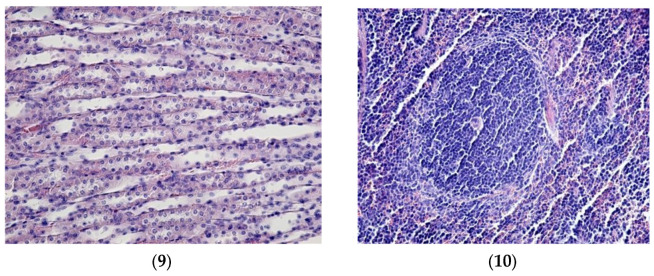
Histopathological findings associated with subacute toxicity of *Calendula arvensis.* Histopathological examination of the liver revealed mild granular–vacuolar degeneration ((**1**), ×20). The vascular structures, including sinusoidal capillaries and the centrilobular vein, showed no histological alterations ((**2**), ×20), while the porto-biliary space appeared normal ((**3**), ×10). Lung tissue exhibited a normal histological architecture across all examined magnifications, including low magnification ((**4**), ×10), intermediate magnification ((**5**), ×20), and high magnification ((**6**), ×40), with preserved alveolar septa and normal pleural structure. The myocardium showed a normal histological appearance without detectable structural abnormalities ((**7**), ×20). Renal examination indicated a normal cortical structure, with intact glomeruli ((**8**), ×20), while the medullary region displayed normal collecting ducts of Bellini ((**9**), ×20). The spleen presented no histopathological changes, with preserved architecture of both white pulp (Malpighian corpuscles) and red pulp ((**10**), ×20).

**Figure 11:**
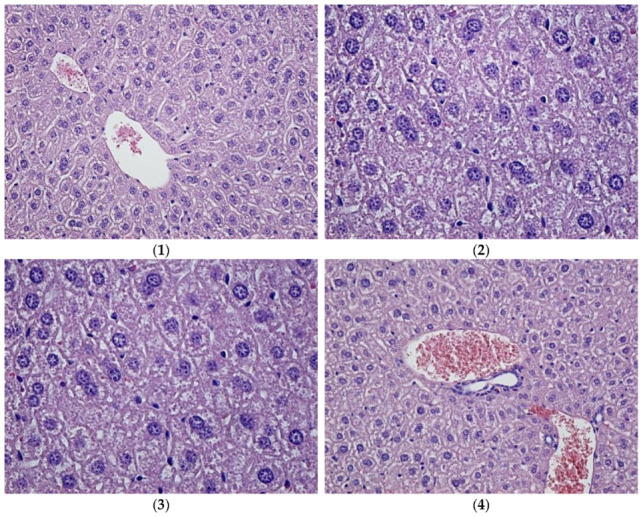

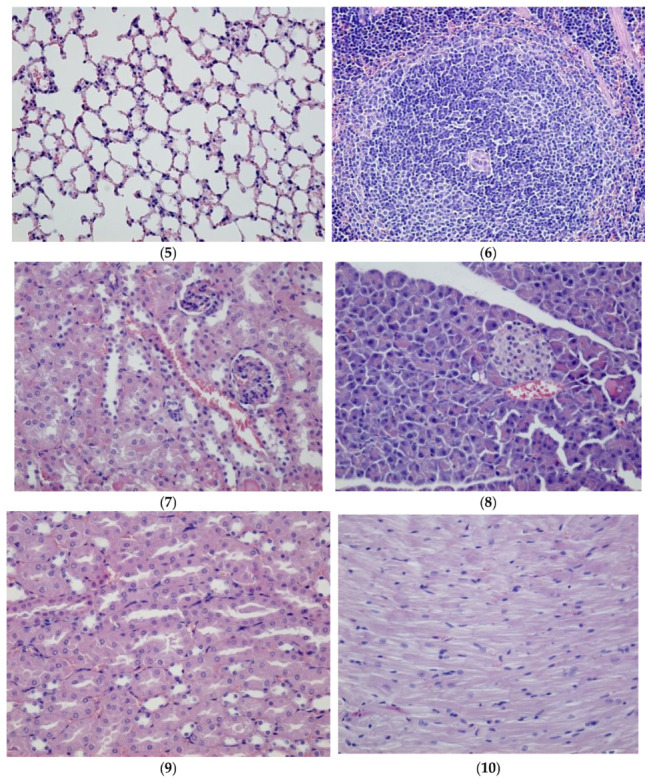
Histopathological findings associated with subacute toxicity of *Polygonum hydropiper.* Histopathological examination of the liver revealed granular–vacuolar degeneration, more pronounced than that observed in mice treated with *Calendula arvensis*, without involvement of the vascular system (**1**). Hepatotoxic lesions were evident, characterized by reduced nuclear visibility, thickening of the cellular membrane, and cytoplasm containing numerous granules and vacuoles ((**2**,**3**), ×40). These alterations were confined to hepatocytes, while vascular structures and the porto-biliary space remained unaffected. No fibrosis or inflammatory reaction was observed ((**4**), ×20). The lung tissue showed no pathological alterations, maintaining a normal histological appearance ((**5**), ×10). The spleen exhibited no histopathological changes, with preserved normal architecture ((**6**), ×20). The pancreas displayed a normal histological structure, with no detectable lesions ((**7**), ×20). Renal examination revealed a normal cortical region ((**8**), ×20) and a normal medullary structure with intact collecting ducts of Bellini ((**9**), ×20). The myocardium showed no signs of degeneration, maintaining normal histological features ((**10**), ×20).

**Figure 12:**
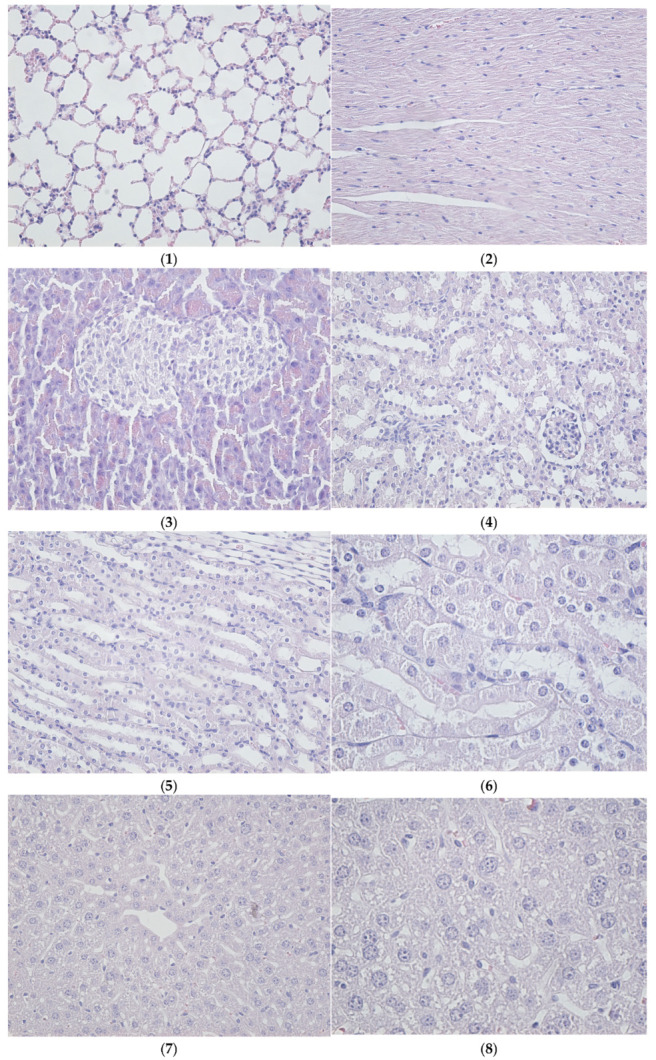

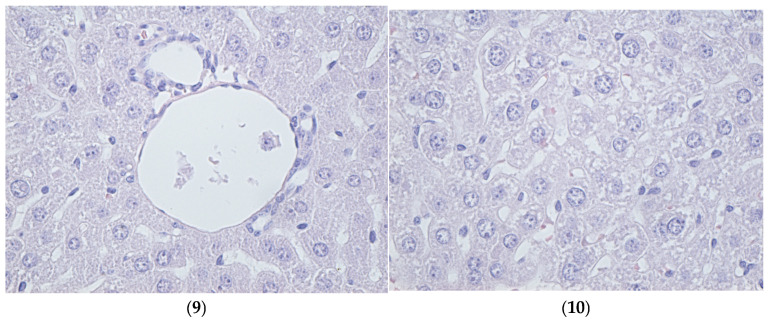
Histopathological findings associated with subacute toxicity of *Agrimonia pilosa.* Histopathological examination of the lung revealed no structural alterations, with a normal tissue architecture ((**1**), ×10). Similarly, the myocardium exhibited a normal histological appearance, without detectable lesions ((**2**), ×20). The pancreas showed structural alterations, particularly at the level of the islets of Langerhans, characterized by disorganization of both glandular and insular parenchyma ((**3**), ×20). Renal histology revealed significant pathological changes, including the presence of vacuoles within glomerular cells ((**4**), ×20). The uriniferous tubules exhibited degenerative changes ((**5**), ×20) and areas of cytolysis, indicative of cellular death, with loss of nuclear integrity ((**6**), ×40). These findings suggest notable nephrotoxic effects. The liver showed degenerative changes, with the centrilobular vein remaining structurally intact ((**7**), ×20), indicating no vascular involvement. However, hepatocytes exhibited marked granular–vacuolar degeneration ((**8**), ×40), along with areas of cytolysis within the hepatic parenchyma. The portal (Kiernan) space appeared normal, without fibrosis or inflammatory infiltration, confirming that the lesions were primarily localized at the level of hepatocytes ((**9**), ×40). The spleen maintained a normal histological structure, with no detectable pathological alterations ((**10**), ×20).

**Figure 13:**
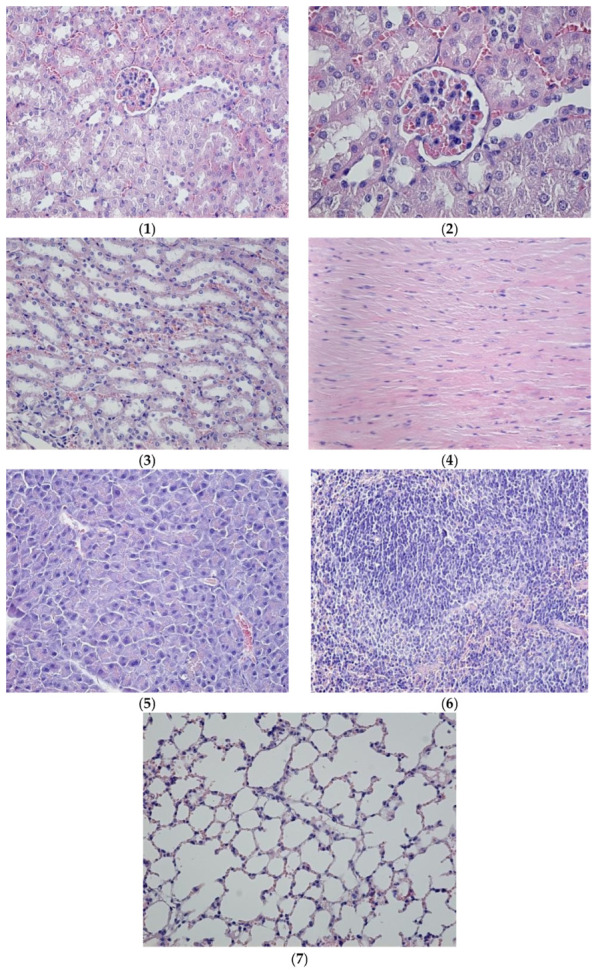
Histopathological findings for control group. Renal histology revealed a normal cortical structure, with glomeruli displaying a typical appearance ((**1**,**2**), ×40). The medullary region was also normal, with well-preserved collecting ducts ((**3**), ×20). The myocardium exhibited a normal histological structure, without detectable abnormalities ((**4**), ×20). The pancreas showed a completely normal architecture, with no structural alterations ((**5**), ×20). The spleen presented a normal lymphoid follicle (Malpighian corpuscle), with preserved organization of the splenic tissue ((**6**), ×20). The lung tissue displayed a normal histological appearance, with intact alveolar structures ((**7**), ×10).

Histological examination of biological tissues was conducted to assess potential adverse effects induced by the subchronic administration of the investigated tinctures (Figure 10, Figure 11, Figure 12 and Figure 13). The histopathological evaluation revealed species-dependent tissue responses following repeated administration of the investigated tinctures. In the *Calendula arvensis* group, only mild hepatic granular–vacuolar degeneration was observed, while the lung, myocardium, kidney, and spleen maintained normal histological architecture. These findings suggest a limited toxicological impact under the tested conditions. In the *Polygonum hydropiper* group, liver examination revealed more evident granular–vacuolar hepatocellular degeneration, suggesting moderate hepatic susceptibility; however, no fibrosis or marked inflammatory infiltrate was observed, and the other examined organs showed relatively preserved morphology. In contrast, the *Agrimonia pilosa* group exhibited more pronounced histopathological changes, including hepatic degenerative lesions, renal structural alterations, and pancreatic disorganization. These findings indicate that repeated administration may induce organ-specific effects, particularly at hepatic, renal, and pancreatic levels, supporting the need for further biochemical and hematological confirmation.

## 3. Discussion

The present findings underline both the preliminary pharmacodynamic potential and the toxicological characteristics of the investigated plant-derived tinctures. The oral glucose tolerance test (OGTT) showed glucose-lowering effects in mice subjected to glucose-induced hyperglycemia, with responses influenced by both dose and time. Notably, *Agrimonia pilosa* exhibited the most consistent reduction in blood glucose levels across the evaluated time points, suggesting a sustained effect under the experimental conditions used. In contrast, *Polygonum hydropiper* and *Calendula arvensis* showed more time-dependent responses, with reductions observed mainly at later intervals. This pattern may indicate differences in the kinetics of bioactive compounds or in the underlying biological processes involved, such as delayed carbohydrate absorption, modulation of glucose metabolism, or enhanced peripheral glucose utilization. However, these mechanisms were not directly investigated and should therefore be interpreted as plausible hypotheses.

From a safety perspective, the acute toxicity assessment indicated a broad safety margin, as no mortality or severe adverse effects were observed even at high doses. These findings support the relative safety of the tested tinctures after single-dose administration. However, repeated administration revealed species-dependent differences in tolerability. *Calendula arvensis* displayed the most favorable subacute safety profile, with no major changes in physiological parameters and only mild, non-progressive hepatic alterations. In contrast, *Polygonum hydropiper* induced more evident hepatocellular changes, mainly granular–vacuolar degeneration. Although these alterations were not accompanied by fibrosis or marked inflammatory infiltration, they may reflect an early adaptive or potentially reversible hepatic response to prolonged exposure. The more pronounced hepatic, renal, and pancreatic alterations observed after *Agrimonia pilosa* administration indicate the need for careful dose optimization and further toxicological evaluation.

The present study supports the growing scientific interest in medicinal plants as complementary sources of bioactive compounds with potential relevance in diabetes management. Ethnobotanical and pharmacological data indicate that many medicinal species traditionally used for glycemic control contain phenolic acids, flavonoids, terpenoids, tannins, and polysaccharides capable of modulating carbohydrate metabolism, oxidative stress, inflammation, and insulin-related pathways [34]. Within this context, the investigated tinctures of *Agrimonia pilosa*, *Calendula arvensis*, and *Polygonum hydropiper* provide a relevant phytopharmaceutical model, as they combine phytochemical richness with measurable antioxidant activity and preliminary glucose-lowering potential.

The results obtained for *Agrimonia pilosa* are consistent with previous evidence showing that this species, belonging to the Rosaceae family, contains a broad spectrum of secondary metabolites, including flavonoids, isocoumarins, triterpenes, phloroglucinol derivatives, tannins, and organic acids [35]. Several compounds reported in *A. pilosa*, such as quercetin derivatives, rutin, hyperoside, agrimonolide, corosolic acid, ursolic acid, and ellagitannins, have been associated in the literature with antioxidant, anti-inflammatory, hepatoprotective, insulin-sensitizing, and α-glucosidase inhibitory effects [35]. Therefore, the glucose-lowering effect observed in the OGTT may be related not to a single constituent, but rather to the cumulative and possibly synergistic action of multiple phytochemical groups.

The importance of polyphenols in *Agrimonia* species is further supported by studies on *Agrimonia eupatoria*, where selective extracts were shown to contain luteolin, quercetin, apigenin, and kaempferol derivatives, together with relevant antioxidant and antimicrobial potential and no marked cytotoxicity in MTS testing [36]. Similarly, hydroethanolic extracts of *A. eupatoria* have been reported to contain gallic and ellagic acids, catechins, hydroxycinnamic acids, and flavonoids such as isoquercitrin, naringenin, and luteolin, while also showing anti-inflammatory and hepatoprotective properties [37]. These findings are relevant for interpreting the present results, since the analyzed tinctures contained polyphenolcarboxylic acids and flavonoids, compounds commonly involved in redox balance and metabolic regulation.

The assignment of phenolic compounds in the present study was based on comparison with reference standards using Rf values and in situ UV spectra. However, TLC combined with UV spectral comparison provides only tentative identification, particularly in complex hydroalcoholic plant extracts containing structurally related phenolic acids and flavonoid derivatives. This limitation was evident for the APH tincture, where the band at Rf 0.27 showed chromatographic behavior close to chlorogenic acid but did not present complete UV spectral overlap with the reference standard. Therefore, this compound was not considered definitively identified as chlorogenic acid and may correspond to a related hydroxycinnamic acid derivative, an ester of caffeic acid, or a co-migrating phenolic compound. By contrast, the bands observed in CAH and PHH showed greater similarity to the chlorogenic acid standard and were therefore described as putatively assigned. Since many phenolic compounds absorb in the UV region around 280 nm and may show partially overlapping spectra, unambiguous structural confirmation requires advanced analytical techniques such as HPLC-DAD, LC-MS/MS, LC-HRMS, or DART-MS.

Several phenolic constituents previously reported in related medicinal plant species, including luteolin, apigenin, isoquercitrin, isorhamnetin, kaempferol, and ferulic acid, were not specifically identified in the present TLC analysis. This non-detection should not be interpreted as confirmation of their absence. It may be related to the limited sensitivity and resolution of TLC, co-migration with other compounds, low concentrations in the analyzed tinctures, matrix effects, the lack of corresponding reference standards during the initial screening, or variability associated with plant material origin, harvesting conditions, and extraction efficiency. Therefore, future studies should include targeted HPLC-DAD, UHPLC-MS/MS, or LC-HRMS analyses using a broader panel of commercially available standards.

The relevance of medicinal plants in diabetes therapy is also supported by broader ethnopharmacological reviews, which emphasize that antidiabetic plant extracts may act through inhibition of carbohydrate-metabolizing enzymes, stimulation of insulin secretion, improvement of insulin sensitivity, modulation of lipid metabolism, and reduction in oxidative damage [38]. In line with this, phytochemicals isolated from medicinal plants have been described as promising candidates for antidiabetic drug discovery, particularly because many of them may act simultaneously on several therapeutic targets rather than through a single mechanism [28]. This multi-target profile is especially relevant in diabetes mellitus, where hyperglycemia, oxidative stress, inflammation, β-cell dysfunction, and insulin resistance interact in a complex pathophysiological network.

The present results are also comparable with experimental data showing that combined plant extracts may produce stronger antidiabetic effects than individual extracts. For example, the combined ethanolic extract of *Syzygium polyanthum* and *Muntingia calabura* leaves showed α-glucosidase inhibition, reduced blood glucose in streptozotocin-induced rats, and demonstrated an LD50 above 2000 mg/kg body weight, indicating both pharmacological efficacy and acceptable acute safety [39]. This supports the idea that complex plant matrices, such as tinctures, may exert biologically meaningful effects through interactions between phenolics, flavonoids, and other secondary metabolites.

The antioxidant activity observed in the present study is particularly relevant because diabetes is associated with increased production of reactive oxygen species, mitochondrial dysfunction, chronic inflammation, and impaired β-cell function. Therefore, extracts rich in antioxidant compounds may help limit oxidative injury and support metabolic homeostasis. Reviews of therapeutic medicinal plants indicate that herbal products may delay the onset of metabolic imbalance and diabetes-related complications, although their efficacy depends strongly on phytochemical composition, dose, experimental model, and duration of administration [40,41]. At the mechanistic level, polyphenolic compounds may contribute to glucose regulation through inhibition of α-amylase and α-glucosidase, reduction in postprandial glucose absorption, enhancement of glucose uptake in insulin-sensitive tissues, improvement of insulin signaling, and protection of pancreatic β-cells against oxidative and inflammatory stress. Some polyphenols may also interfere with human amylin aggregation, a process implicated in β-cell toxicity in type 2 diabetes [42]. However, these mechanisms were not directly tested in the present study and should be regarded as literature-supported hypotheses.

The relationship between the Rosaceae family and antidiabetic activity is further supported by studies in streptozotocin-induced diabetic rats, where isolated fractions from Rosaceae plants improved insulin secretion and contributed to pancreatic β-cell repair [43]. This is relevant for *Agrimonia pilosa*, as the stronger glucose-lowering response observed at higher doses may reflect not only delayed glucose absorption but also possible pancreatic or insulin-related mechanisms. Moreover, recent data on Rosaceae medicinal plants have emphasized their richness in ellagitannins and other polyphenols, compounds that may positively influence lipid metabolism, inflammation, and metabolic risk factors associated with type 2 diabetes [44].

The results obtained for *Calendula arvensis* may be interpreted within the broader pharmacological context of the Asteraceae family. Asteraceae species are widely used in ethnomedicine and have been reported to possess antioxidant, hepatoprotective, anti-inflammatory, wound-healing, and antidiabetic effects [45]. Several members of this family contain polysaccharides, flavonoids, phenolic acids, terpenoids, and other compounds that may improve glucose metabolism and reduce oxidative stress. In addition, recent reviews on *Silybum marianum* and *Brachylaena discolor* highlight the potential of Asteraceae species in type 2 diabetes management, while also emphasizing the need for more complete long-term toxicity evaluation [46]. This is relevant to the present findings, where *Calendula arvensis* showed moderate biological activity but a comparatively more favorable subacute histological profile.

For *Polygonum hydropiper*, the present phytochemical and antioxidant findings agree with previous work showing that water pepper is a relevant source of polyphenolic compounds, especially flavonoids and phenolic acids, and that extraction solvent and method strongly influence the yield and antioxidant potential of the obtained extracts [47]. This is consistent with the current use of 70% ethanol tinctures, which are suitable for extracting both moderately polar phenolic acids and flavonoid derivatives. The higher total polyphenol and flavonoid content observed for *Polygonum hydropiper* in the present study may therefore explain its marked antioxidant potential. The antidiabetic relevance of the Polygonaceae family is further supported by studies on *Polygonum maritimum*, in which methanolic leaf and root extracts showed strong antioxidant activity and α-glucosidase inhibition, while dichloromethane extracts also demonstrated anti-inflammatory effects in stimulated macrophages [48]. These findings suggest that species of the genus *Polygonum* may act through combined antioxidant, anti-inflammatory, and enzyme-inhibitory mechanisms. Consequently, the glucose-lowering effect observed for *Polygonum hydropiper* may be partly explained by delayed carbohydrate digestion and reduced postprandial glycemic excursions.

Overall, the glucose-lowering effects observed in the OGTT may be partly related to the phenolic profile of the tinctures. Hydroxycinnamic acids, caffeic/chlorogenic acid-related derivatives, and flavonoid-type compounds have been reported to influence glucose metabolism through several mechanisms, including inhibition of α-glucosidase and α-amylase, modulation of insulin signaling pathways, improvement of oxidative stress, and protection of pancreatic β-cells. Nevertheless, these mechanisms were not directly investigated in the present work. Therefore, the relationship between phenolic composition, antioxidant activity, and hypoglycemic potential should be regarded as a plausible hypothesis rather than a demonstrated mechanism. Further studies using enzyme inhibition assays, insulin signaling markers, diabetic animal models, and targeted phytochemical profiling are required to identify the compounds most directly involved in the glucose-lowering effects.

Several limitations should be acknowledged. First, the absence of a positive control drug, such as metformin, glibenclamide, or acarbose, limits the pharmacological interpretation of the hypoglycemic evaluation. Therefore, the observed glucose-lowering effects should be interpreted as preliminary evidence of hypoglycemic potential rather than definitive antidiabetic efficacy. Second, the toxicity assessment was not complemented by serum biochemical or hematological parameters. Although histopathological examination provided useful information regarding potential organ-specific effects, the lack of liver, kidney, and pancreatic biomarkers limits the strength of the toxicological conclusions. Future studies should include ALT, AST, ALP, bilirubin, creatinine, BUN, electrolytes, insulin, amylase, lipase, and complete hematological profiles. Third, the phytochemical characterization was based mainly on TLC and spectrophotometric methods rather than advanced chromatographic techniques. Therefore, the phytochemical profile should be interpreted as preliminary and comparative. Future studies should include advanced chromatographic and mass spectrometric analyses for comprehensive profiling and quantification of bioactive compounds.

## 4. Materials and Methods

### 4.1. Establishment of the Identity, Purity, and Quality of the Plant Material

The plant material used in this study was obtained from species cultivated in the “Alexandru Buia” University Botanical Garden of Craiova. Voucher specimens, collected at different time intervals between 2015 and 2018, are preserved in the Collection of the Pharmacognosy Laboratory, Faculty of Pharmacy, University of Medicine and Pharmacy of Craiova. The analyzed plant material included *Agrimonia pilosa* Ledeb. from the Rosaceae family, represented by *Agrimoniae pilosae herba* (APH), *Calendula arvensis* L. from the Asteraceae family, represented by *Calendulae arvensis herba* (CAH), and *Polygonum hydropiper* L. from the Polygonaceae family, represented by *Polygoni hydropiperis herba* (PHH). The common names recorded for *Calendula arvensis* L. were Hilimică and Filimică, while *Polygonum hydropiper* L. was known as Piperul bălţii and Dintele dracului. The identity, purity, and quality of the plant products were established based on specific analytical procedures performed in accordance with specialized methodologies [49,50,51,52].

### 4.2. Preparation of Tinctures by Simple Percolation According to F.R. X

The tinctures were obtained by simple percolation, using a plant material-to-solvent ratio of 1:5 and 70% ethanol as extraction solvent. The plant materials were naturally dried and subsequently pulverized using an electric grinder until the particle size corresponded to sieve IV. For each gram of plant material, 0.5 mL of diluted ethanol was used for moistening, and the mixture was maintained at room temperature for three hours in tightly closed containers.

Following this step, the plant materials were passed through sieve I and introduced into the percolator by gentle pressing. The solvent was gradually added until it began to flow through the lower outlet, while a thin layer of liquid remained above the plant material. After closing the outlet, the system was allowed to stand for 24 h, after which the percolation process was initiated. The percolation rate was adjusted to obtain 1.5 g of extractive solution per gram of plant material within 24 h.

Throughout the two-week extraction period, the plant material remained continuously covered with solvent. Percolation was stopped after obtaining 500 mL of each tincture. The extracts were then allowed to stand for seven days at 5–10 °C, filtered, and stored in 100 mL amber glass containers, tightly closed and protected from light at room temperature [51,53,54].

### 4.3. Organoleptic Characterization

The obtained tinctures were clear, colored liquids, with odor and taste characteristic of both the plant material and the hydroalcoholic solvent. Upon dilution with water, the solutions became opalescent or turbid, in agreement with pharmacopoeial specifications [51,53,54].

### 4.4. Determination of Relative Density According to F.R. X

The relative density was defined as the ratio between the mass of a given volume of substance at 20 °C and the mass of an equal volume of water at the same temperature. Measurements were carried out using a pycnometer. The mass of the empty pycnometer, the pycnometer filled with water, and the pycnometer filled with the analyzed sample were determined, and the relative density was calculated using the following relation:

$$d^{20}=\frac{m_{liquid}}{m_{H2O}}$$

where $m_{liquid}$ represents the mass of the analyzed liquid and $m_{H2O}$ represents the mass of water. The precision of the determination was expressed to four decimal places [51].

### 4.5. Determination of Refractive Index According to F.R. X

The refractive index relative to air was defined as the ratio between the speed of light in air and in the analyzed sample and was calculated according to the relation n=sinα/sinβ. Measurements were performed using an Abbé refractometer.

### 4.6. Quality Requirements According to F.R. X

The iron content was required not to exceed 0.001%. For this purpose, 3 g of tincture was evaporated to dryness, and the residue was calcined with concentrated sulfuric acid. The resulting solution was compared with a Fe^3+^ standard solution according to pharmacopoeial procedures.

Similarly, the heavy metal content was limited to a maximum of 0.001%, determined by comparison with a Pb^2+^ standard solution after calcination and processing of the residue.

The alcohol concentration was determined by distillation of ethanol followed by measurement of the relative density of the hydroalcoholic distillate. The alcohol concentration was obtained from alcoholometric tables and calculated using the formula:

$$\%alcohol=100\times \frac{c}{m_{t}}$$

where c represents the alcohol concentration corresponding to the measured density and $m_{t}$ represents the mass of the analyzed tincture [51].

The residue on evaporation was determined by drying 10 g of tincture at 105 °C for three hours, and the result was expressed relative to 100 g of sample [50,52,53].

### 4.7. Thin-Layer Chromatographic Analysis of Flavonoids and Polyphenolcarboxylic Acids

Thin-layer chromatography was employed for the separation and identification of flavonoids and polyphenolcarboxylic acids, including caffeic acid and chlorogenic acid. The method relied on differential migration on silica gel plates using a selective solvent system, followed by identification through comparison with reference standards [54,55,56,57,58].

Chromatographic analyses were performed on silica gel G 60 plates with fluorescence indicator F254 (Merck, Darmstadt, Germany). The mobile phase consisted of ethyl acetate–formic acid–methanol–water (15:1:0.1:1), and detection was carried out under UV light at 254 nm and 280 nm using a CAMAG TLC Scanner 3 system (CAMAG, Muttenz, Switzerland). The selected TLC conditions were used as a preliminary screening approach for the qualitative evaluation of phenolic acids and flavonoid-type compounds in hydroalcoholic tinctures. The mobile phase was chosen based on its suitability for separating relatively polar phenolic constituents on silica gel plates. However, due to the high polarity of several polyphenolic compounds and their strong interaction with the stationary phase, partial retention near the origin may occur. Therefore, the TLC results were interpreted as indicative phytochemical fingerprints rather than definitive compound identification. Alternative solvent systems with higher elution strength, gradient HPTLC approaches, two-dimensional TLC, or HPLC/LC-MS-based methods may improve resolution and identification accuracy.

### 4.8. Quantitative Determination of Phenylpropanoid Compounds

The phenylpropanoid compounds were quantified spectrophotometrically using the Arnow method, based on the formation of colored oximes [49,50,51]. The calibration curve for caffeic acid showed very good linearity, described by the regression equation:

$$y=0.0081x+0.0175,R^{2}=0.9887$$

Absorbance was measured at 510 nm, and the results were calculated based on the difference between sample and blank readings.

### 4.9. In Vitro Analysis of Antioxidant Capacity

The total polyphenol content was determined using the Folin–Ciocâlteu method [59,60,61,62]. The calibration curve constructed using gallic acid as a reference standard showed good linearity, with the regression equation:

$$y=0.0036x+0.1987,R^{2}=0.9823$$

The total flavonoid content was determined spectrophotometrically using aluminum chloride, with quercetin as a reference compound. The calibration curve demonstrated excellent linearity, described by the equation:

$$y=0.0095x-0.0128,R^{2}=0.9901$$

All measurements were performed under controlled experimental conditions, and the results were expressed as gallic acid equivalents and quercetin equivalents, respectively.

### 4.10. Determination of Acute Toxicity in Mice

According to the Globally Harmonized System (GHS), acute toxicity is defined as “the adverse effects occurring following oral or dermal administration of a single dose of a substance, or multiple doses given within 24 h, or an inhalation exposure of 4 h”. Oral administration represents the most commonly used route for assessing acute systemic toxicity, and the results are typically expressed as LD_50_ values (approximate median lethal dose).

Although the rat is generally the preferred species for acute toxicity testing via oral or inhalation routes, in the present study mice were selected in order to ensure consistency with subsequent experimental protocols involving streptozotocin-induced diabetes, which were also performed in mice. Typically, the group size for acute and subacute toxicity studies consists of five animals; however, in the present study, a larger number of animals was used to improve the robustness of the observations.

The estimation of acute toxicity for classification purposes is based on LD_50_ or LC_50_ values when available. In the case of mixtures, toxicity may be derived from known LD_50_/LC_50_ values of individual components or, alternatively, assessed experimentally for the entire mixture. The classification of substances into acute toxicity categories is based on dose ranges corresponding to five levels of toxicity, expressed in mg/kg body weight for oral administration.

Category 5 includes substances with relatively low acute toxicity, which may still pose risks under certain conditions, particularly for vulnerable populations. These substances typically have LD_50_ values within the range of 2000–5000 mg/kg body weight. Due to ethical considerations regarding animal welfare, testing within this category is generally discouraged and should only be conducted when there is strong justification for its relevance to human health protection [63].

The experimental animals used in this study were male CD1 mice, aged 6–8 weeks, with body weights ranging between 20 and 30 g, ensuring that inter-individual variation did not exceed ±20%. The animals were housed in standard laboratory cages and allowed to acclimatize for seven days prior to the experiment. Environmental conditions were maintained at a temperature of 22 ± 3 °C and a relative humidity of 30–70%, with a controlled light–dark cycle of 12 h light and 12 h darkness.

For the acute toxicity assessment, five experimental groups were established, each consisting of ten male mice. Each group was further subdivided into five subgroups of two animals each, which received a single oral dose of the investigated tinctures by gavage, at dose levels of 1, 2, 3, 4, and 5 g/kg body weight. A control group was also included, receiving physiological saline administered by gavage.

The administered volume did not exceed 50 mL/kg body weight. Prior to dosing, the animals were subjected to overnight fasting starting at 20:00. On the following morning, between 09:00 and 10:00, the animals were weighed and the test substance was administered as a single dose adjusted to body weight. Food and water were provided ad libitum two hours after administration.

Following treatment, the animals were observed clinically for a period of 24 h, with particular attention given to behavioral, neurological, and physiological parameters indicative of potential toxic effects (Table 10) [64].

The monitored parameters included both signs associated with lethality, such as convulsions, apnea, and coma, as well as behavioral changes, including alterations in locomotion, eyelid position, levels of sedation or agitation, excessive salivation, sleep duration, the appearance of fur and mucous membranes, tremors of the extremities, photophobia, and respiratory rate. In acute toxicity studies, adverse effects are typically observed within the first 24 h following administration of the tested substance.

### 4.11. Subacute Toxicity Assessment of the Investigated Plant Tinctures

Subacute toxicity testing involves the evaluation of general toxic effects resulting from repeated daily exposure of laboratory animals to a given substance over a defined period of time. Such studies provide essential information regarding the cumulative effects of compounds and their potential impact on physiological and biochemical functions.

The assessment of subacute toxicity encompasses a wide range of parameters, including changes in body weight, absolute and relative organ weights, alterations in clinical biochemistry, urinalysis, and hematological parameters, as well as functional disturbances affecting the nervous system and other organs. In addition, microscopic examination of tissues allows the identification of pathological changes at the organ level. The subacute toxicity study was conducted on male CD1 mice, aged 4–6 weeks and weighing between 20 and 30 g. A total of four experimental groups were established, each consisting of four animals. Three groups received tinctures obtained from *Calendula arvensis* L., *Polygonum hydropiper* L., and *Agrimonia pilosa* Ledeb., while one group served as the control and received physiological saline.

The tinctures were administered orally by gavage at a dose of 400 mg/kg body weight, once daily, between 09:00 and 10:00, for a period of 14 consecutive days. Following administration, the animals were monitored daily throughout the study for signs of toxicity or mortality.

Observations included food and water intake, body weight evolution, survival rate, and the presence of any clinical signs indicative of general toxicity. At the end of the 14-day experimental period, all animals were sacrificed in accordance with animal welfare regulations [65,66,67].

### 4.12. Histopathological Aspects Following Animal Sacrifice After 14 Days of Tincture Administration

After 14 days of administration of the tinctures obtained from the investigated plant species, the animals were sacrificed in accordance with established ethical guidelines for animal protection. Organs of interest were carefully collected and processed following standard histopathological protocols. Tissue samples were obtained from the lungs, liver, kidneys, myocardium, spleen, and pancreas for each experimental group.

The collected tissues were subjected to conventional paraffin-embedding procedures, followed by hematoxylin–eosin staining. The preparation of histological sections involved several sequential steps designed to preserve tissue morphology and ensure optimal visualization under light microscopy.

Initially, tissue dehydration was performed to completely remove water from the biological samples. This process involved immersion in increasing concentrations of ethanol, starting with 75% ethanol (twice), followed by 90% ethanol (twice), and finally absolute ethanol (twice). Subsequently, tissue clarification was carried out using xylene, which ensured the removal of residual alcohol and enhanced tissue transparency.

Paraffin infiltration was then performed using molten paraffin, allowing for uniform penetration into the tissue and providing the necessary consistency for obtaining thin sections. The embedding process involved placing the tissues in plastic molds filled with molten paraffin, resulting in solid paraffin blocks.

The paraffin blocks were sectioned using a microtome to obtain slices with a thickness of approximately 5 μm. These sections were carefully mounted on clean, degreased glass slides and allowed to dry prior to staining.

Hematoxylin–eosin staining was subsequently performed through a series of standard steps, including deparaffinization, rehydration, staining with hematoxylin, differentiation with hydrochloric acid, treatment with lithium carbonate solution, counterstaining with eosin, dehydration with 70% ethanol followed by absolute ethanol, and final clarification in xylene. After each step, the slides were briefly rinsed with distilled water to ensure proper reagent removal.

The hematoxylin–eosin staining technique enabled clear differentiation of tissue components based on their staining properties. Cell nuclei appeared intensely blue-violet, while the cytoplasm exhibited a lighter violet coloration. Collagen fibers were stained pale pink, whereas elastic and reticular fibers were not specifically highlighted by this method [68,69,70].

### 4.13. Determination of the Effective Dose of the Investigated Tinctures Using the Oral Glucose Tolerance Test in Normoglycemic Mice

The experiment was conducted on seven groups of male CD1 mice, aged 4–6 weeks. Each group consisted of nine animals, except for the control group, which received physiological saline. The experimental groups were further subdivided into three subgroups of three animals each, allowing the evaluation of three different dose levels for each tested tincture.

The animals in each experimental group were pretreated with the same tincture administered orally by gavage, but at three different doses: 100 mg/kg body weight, 150 mg/kg body weight, and 200 mg/kg body weight. The highest administered dose (200 mg/kg body weight) corresponded to approximately one-tenth of the estimated LD_50_ value for the investigated tinctures.

The control group received physiological saline, while the treatment groups received tinctures prepared from *Polygonum hydropiper* L. (*Polygoni hydropiperis herba*, PHH), *Calendula arvensis* L. (*Calendulae arvensis herba*, CAH), and *Agrimonia pilosa* Ledeb. (*Agrimoniae pilosae herba*, APH).

The experimental protocol was carried out within a single day. Initially, fasting blood glucose levels were measured (baseline glycemia, a jeun). Subsequently, the tinctures were administered by gavage in a volume of 0.3 mL. After 30 min, a glucose load of 2 g/kg body weight was administered orally.

Blood glucose levels were then determined at 30, 60, 90, and 120 min following glucose administration, allowing the assessment of glucose tolerance and the potential hypoglycemic effect of the tested tinctures.

Glycemia was measured using an Accu-Chek Active glucometer, based on a drop of blood collected from the tail vein. The obtained data were statistically processed by calculating the mean values ± standard deviation for each subgroup and comparing them with the control group.

## 5. Conclusions

The present study provides preliminary evidence that hydroalcoholic tinctures of *Agrimonia pilosa*, *Calendula arvensis*, and *Polygonum hydropiper* contain phenolic compounds and flavonoids and exhibit antioxidant activity and glucose-lowering potential in normoglycemic mice. However, due to the absence of diabetic animal models, positive pharmacological controls, detailed LC-MS-based phytochemical characterization, and biochemical toxicity markers, these findings should be interpreted cautiously. Further studies are required to confirm their mechanisms of action, safety profile, and potential therapeutic relevance in diabetes management. From a phytochemical perspective, the preparation of the tinctures by simple percolation, in accordance with pharmacopoeial standards, ensured the extraction of a wide range of bioactive compounds. Qualitative and quantitative analyses confirmed the presence of flavonoids and polyphenolcarboxylic acids, including chlorogenic and caffeic acids, identified through thin-layer chromatography and spectrophotometric methods. The significant content of these compounds supports the antioxidant capacity of the extracts, as demonstrated by the in vitro evaluation of total polyphenol and flavonoid levels, suggesting their potential role as natural sources of bioactive molecules with hypoglycemic and antioxidant properties.

The assessment of acute toxicity indicated that all investigated tinctures can be classified within category 5 according to the United Nations Globally Harmonized System of Classification and Labelling of Chemicals (2011), suggesting a low level of acute toxicity at therapeutically relevant doses. No severe adverse effects or mortality were observed within the monitored period, supporting their relative safety following single-dose administration.

In contrast, subacute toxicity studies revealed distinct organ-specific effects depending on the plant species. The tincture obtained from *Calendula arvensis* L. did not induce significant toxicological alterations at a dose of 400 mg/kg body weight/day, as no notable changes were observed in behavior, food and water intake, or body weight. The tincture derived from *Polygonum hydropiper* L. exhibited moderate hepatotoxicity, characterized by structural alterations in hepatocytes, without fibrosis or inflammatory processes, while physiological parameters such as food intake, water consumption, and body weight remained unaffected. More pronounced effects were observed for the tincture of *Agrimonia pilosa* Ledeb., which induced hepatic and renal histopathological lesions, along with structural disorganization of the pancreas. These changes were associated with a statistically significant reduction in food intake, although body weight and water consumption were not significantly altered over the experimental period.

Histopathological examination confirmed the presence of organ-specific lesions, particularly at the hepatic level, where degenerative changes in hepatocytes were identified, supporting the biochemical and toxicological findings. Renal and pancreatic alterations observed in certain experimental groups further emphasize the importance of dose-dependent safety evaluation for plant-derived extracts intended for therapeutic use. The oral glucose tolerance test performed on normoglycemic mice demonstrated dose-dependent effects of the investigated tinctures on glucose metabolism. The selected doses for further chronic experimental studies were established as 100 mg/kg body weight for *Polygonum hydropiper*, 150 mg/kg body weight for *Calendula arvensis*, and 200 mg/kg body weight for *Agrimonia pilosa*, based on their pharmacodynamic response and safety profile.

Overall, the findings of this study highlight the dual nature of plant-derived tinctures, combining beneficial antioxidant and hypoglycemic properties with potential organ-specific toxicity at higher or prolonged doses. These results underline the necessity for careful dose optimization and further pharmacological investigations prior to clinical application.

## Figures and Tables

**Figure 1:**
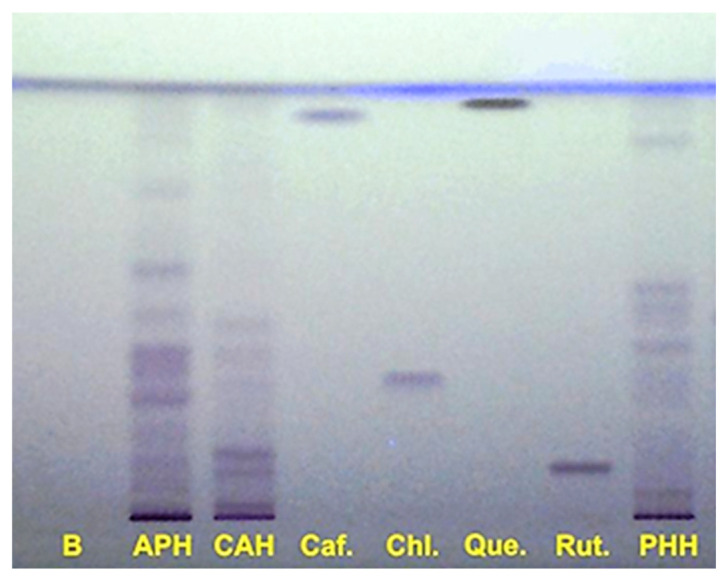
Chromatogram of polyphenolic compounds in the analyzed tinctures (UV detection at λ = 254 nm, without derivatization). From left to right, the first band corresponds to the application-free zone (background, B), followed by two applications (2 μL each) representing the tinctures APH and CAH. Bands 4–7 correspond to four applications (2 μL each) of reference standards: caffeic acid (Caf.), chlorogenic acid (Chl.), quercetin (Que.), and rutin (Rut.). Band 8 (2 μL) corresponds to the tincture PHH. APH—*Agrimoniae pilosae herba*; CAH—*Calendulae arvensis herba*; PHH—*Polygoni hydropiperis herba*.

**Figure 2:**
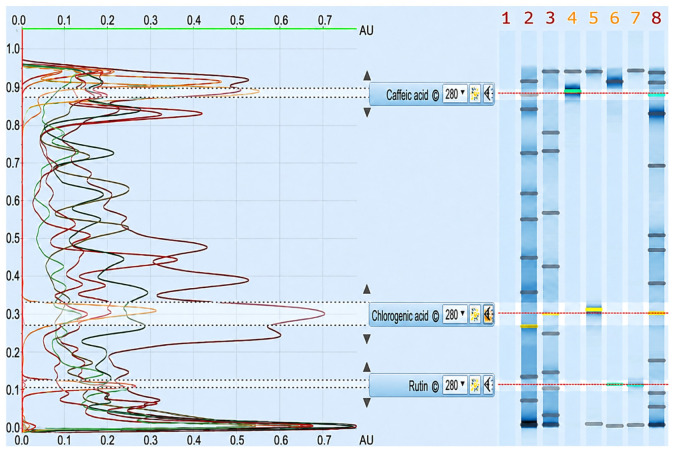
Densitogram of polyphenolic compounds in the analyzed tinctures (UV detection at λ = 280 nm, without derivatization). 1, 2, 3 and 8 = tincture sample track; 4–7 = reference standard track. Red labels indicate tincture sample tracks, while orange labels indicate reference standard tracks.

**Figure 3:**
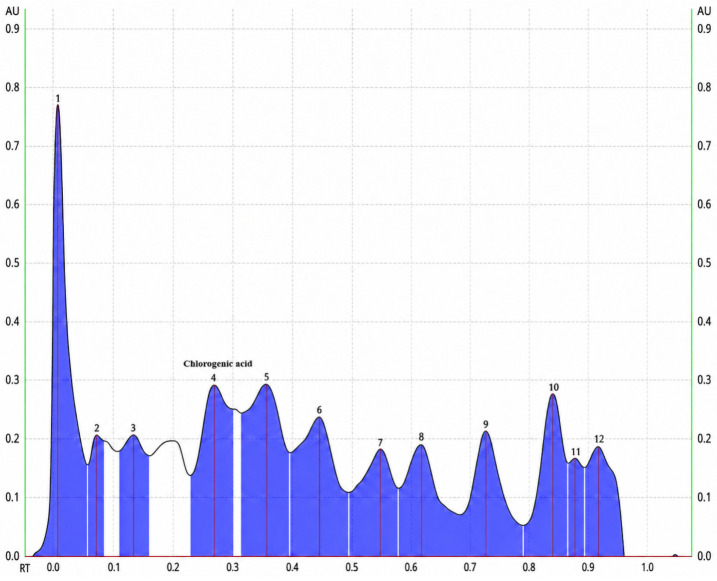
Densitogram of polyphenolic compounds separated from the APH tincture (UV detection at λ = 280 nm, without derivatization).

**Figure 4:**
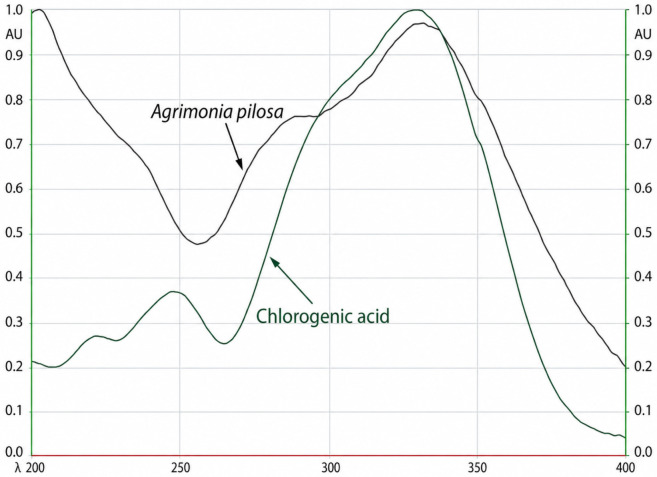
In situ UV spectrum of the chlorogenic acid standard compared with the major compound separated from the APH tincture at Rf 0.27. The incomplete spectral overlap indicates that this band cannot be definitively assigned to chlorogenic acid and may correspond to a structurally related hydroxycinnamic acid derivative or to a co-migrating phenolic compound. UV detection at λ = 280 nm, without derivatization.

**Figure 5:**
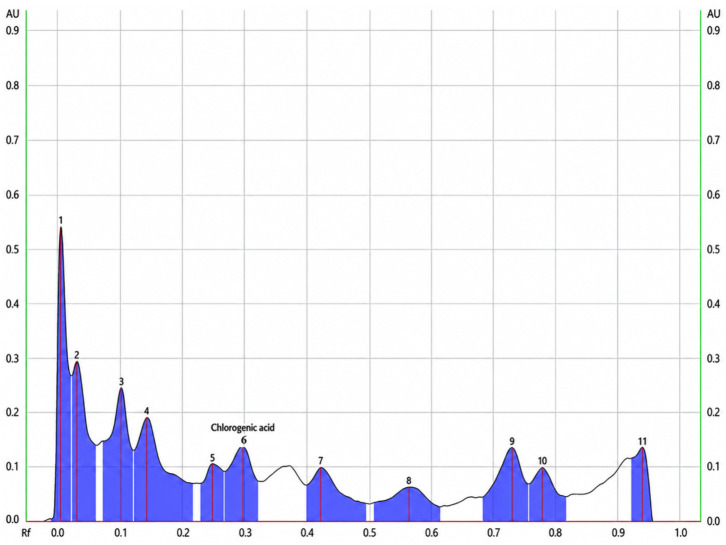
Densitogram of polyphenolic compounds separated from the CAH tincture (UV detection at λ = 280 nm, without derivatization).

**Figure 6:**
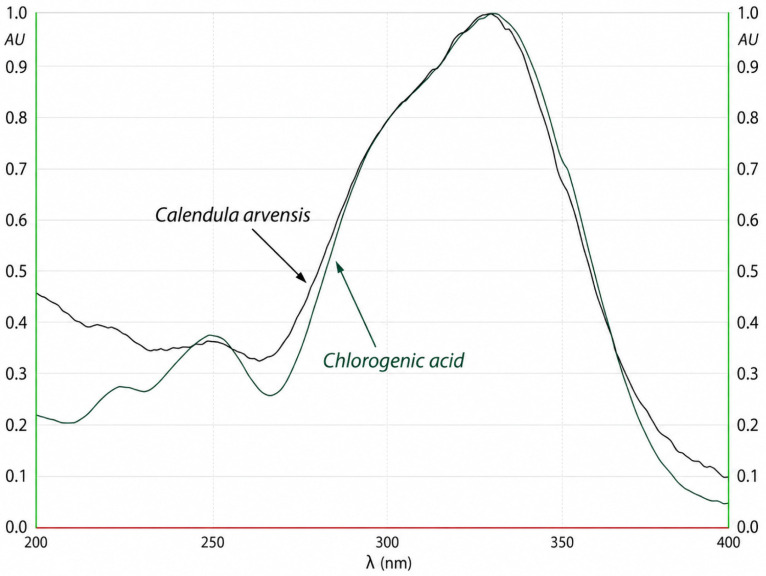
In situ UV spectrum of chlorogenic acid standard compared with chlorogenic acid separated from the CAH tincture (UV detection at λ = 280 nm, without derivatization).

**Figure 7:**
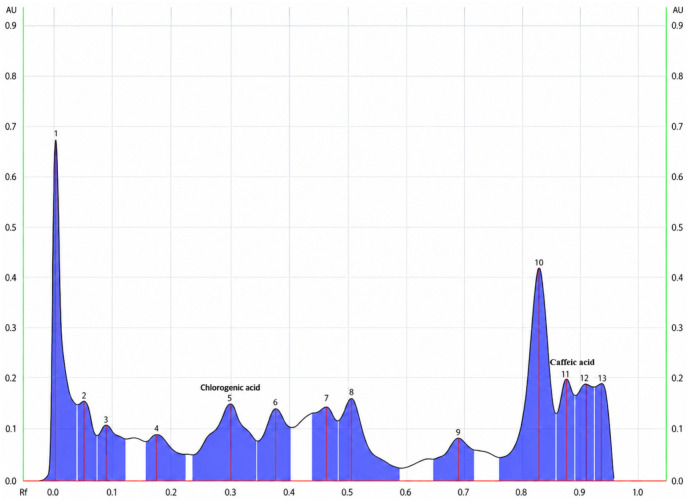
Densitogram of polyphenolic compounds separated from the PHH tincture (UV detection at λ = 280 nm, without derivatization).

**Figure 8:**
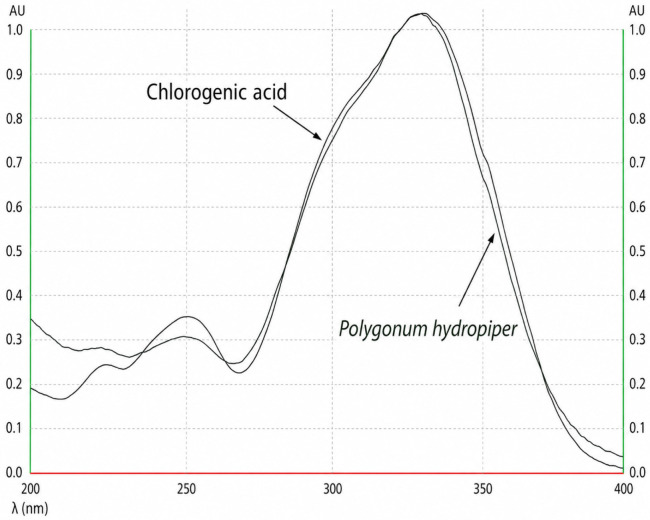
In situ UV spectrum of the chlorogenic acid standard compared with chlorogenic acid separated from the PHH tincture (UV detection at λ = 280 nm, without derivatization).

**Figure 9:**
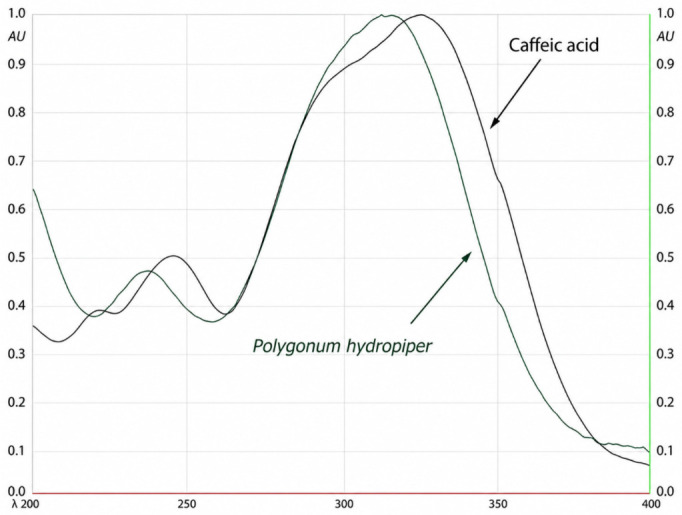
In situ UV spectrum of the caffeic acid standard compared with caffeic acid separated from the PHH tincture (UV detection at λ = 280 nm, without derivatization).

**Table 1:** Physicochemical characteristics of hypoglycemic tinctures.

Parameter	*Agrimoniae pilosae herba*	*Calendulae arvensis herba*	*Polygoni hydropiperis herba*
Appearance	Clear liquid	Clear liquid	Clear liquid
Color	Yellow-orange	Brownish-green	Yellowish-green
Odor	Characteristic, slightly aromatic	Characteristic, slightly aromatic	Characteristic, slightly aromatic
Taste	Slightly bitter, burning	Slightly bitter, burning	Slightly bitter, burning
Relative density	0.9542	0.9675	0.9513
Refractive index	1.3652	1.3644	1.3648
Iron [%]	Not detected	Not detected	Not detected
Heavy metals [%]	Not detected	Not detected	Not detected
Alcohol content [% m/m]	67.55	68.25	67.15
Residue on evaporation [%]	6.85	5.15	5.65
Qualitative analysis (TLC)	Flavonoids, polyphenolcarboxylic acids	Flavonoids, polyphenolcarboxylic acids	Flavonoids, polyphenolcarboxylic acids
Quantitative analysis (VIS spectrophotometry) phenylpropanoids [mg/mL]	0.39	0.14	0.42

**Table 2:** Results obtained from the thin-layer chromatographic (TLC) analysis of polyphenolic compounds in the APH tincture.

Peak No.	Rf Max.	AU	Area [%]	Remarks
1.	0.005	0.025486255	15.72	A major compound with chromatographic behavior close to chlorogenic acid was observed at Rf 0.27. However, because the UV spectrum did not fully overlap with the chlorogenic acid standard, this assignment remains tentative. The band may correspond to a chlorogenic acid-related hydroxycinnamic derivative or to a co-migrating phenolic compound.
2.	0.07	0.005245104	3.24	
3.	0.13	0.010126123	6.25	
4.	0.27	0.017210919	10.62	
5.	0.36	0.02054452	12.67	
6.	0.45	0.017962405	11.08	
7.	0.55	0.012116617	7.48	
8.	0.62	0.013210909	8.15	
9.	0.73	0.013235793	8.16	
10.	0.84	0.012967231	8	
11.	0.88	0.004128496	2.55	
12.	0.92	0.009853842	6.08	

**Table 3:** Results obtained from the thin-layer chromatographic (TLC) analysis of polyphenolic compounds in the CAH tincture.

Peak No.	Rf Max.	AU	Area [%]	Remarks
1.	0.005	0.009486685	12.74	Chlorogenic acid was putatively identified in the CAH tincture at an Rf value of 0.29, with a measured concentration of 276 μg/mL.
2.	0.03	0.008872998	11.92	
3.	0.1	0.008995431	12.08	
4.	0.14	0.011070782	14.87	
5.	0.24	0.003737564	5.02	
6.	0.29	0.006053478	8.13	
7.	0.42	0.006171309	8.29	
8.	0.56	0.004996638	6.71	
9.	0.73	0.00697234	9.36	
10.	0.78	0.00433116	5.82	
11.	0.94	0.00376871	5.06	

Abbreviations: TLC—thin-layer chromatography; CAH—*Calendulae arvensis herba*; AU—area unit.

**Table 4:** TLC analysis results of polyphenolic compounds identified in the PHH tincture.

Peak No.	Rf Max.	AU	Area [%]	Remarks
1.	0.005	0.014314995	14.33	Chlorogenic acid (Rf 0.29) and caffeic acid (Rf 0.88) were putatively identified in the PHH tincture, with concentrations of 512.5 μg/mL and 160 μg/mL, respectively.
2.	0.05	0.004298898	4.3	
3.	0.09	0.004780986	4.78	
4.	0.17	0.004674227	4.68	
5.	0.29	0.01123573	11.24	
6.	0.38	0.006563525	6.57	
7.	0.46	0.005787142	5.79	
8.	0.51	0.008570687	8.58	
9.	0.69	0.004465764	4.47	
10.	0.83	0.019092185	19.1	
11.	0.88	0.005612008	5.62	
12.	0.91	0.005550216	5.56	
13.	0.94	0.004976997	4.98	

**Table 5:** In vitro antioxidant activity of tinctures with hypoglycemic potential.

Tincture (Diluted 1:5 with Distilled Water)	In Vitro Antioxidant Activity	
	Total Polyphenol Content [mg/L GAE]	Total Flavonoid Content [mg/L QE]
*Agrimoniae pilosae herba* (APH)	280.58 ± 5.57	138.29 ± 2.76
*Calendulae arvensis herba* (CAH)	97.05 ± 1.94	113.52 ± 2.27
*Polygoni hydropiperis herba* (PHH)	328.35 ± 6.08	151.65 ± 3.03

Abbreviations: GAE—gallic acid equivalent; QE—quercetin equivalent.

**Table 6:** Determination of acute toxicity for the tinctures of *Calendula arvensis*, *Polygonum hydropiper*, and *Agrimonia pilosa* at doses of 6, 7, 8, and 9 g/kg body weight.

Tincture Type	Mouse	Tincture Dose	Body Weight (g), Pre-Administration	Body Weight (g), 24 h Post-Administration	Blood Glucose (mg/dL), Pre-Administration	Blood Glucose (mg/dL), 24 h Post-Administration
Tincture dose of *Calendula arvensis*	1.	6 g/kg b.w.	24	23	94	110
	2.	6 g/kg b.w.	32	31.5	85	100
	3.	7 g/kg b.w.	30	29	75	95
	4.	7 g/kg b.w.	29	28	81	97
	5.	8 g/kg b.w.	29	28	92	95
	6.	8 g/kg b.w.	28.5	27	66	92
	7.	9 g/kg b.w.	31.5	29	68	99
	8.	9 g/kg b.w.	32	32	63	92
Tincture dose of *Polygonum hydropiper*	1.	6 g/kg b.w.	30.5	29	80	71
	2.	6 g/kg b.w.	30	28.5	47	53
	3.	7 g/kg b.w.	30.5	29	77	75
	4.	7 g/kg b.w.	31	29.5	112	97
	5.	8 g/kg b.w.	33.5	32	92	85
	6.	8 g/kg b.w.	31.5	29.5	89	36
	7.	9 g/kg b.w.	26.5	24.5	89	107
	8.	9 g/kg b.w.	30.5	29	109	117
Tincture dose of *Agrimonia pilosa*	1.	6 g/kg b.w.	31	30.5	89	94
	2.	6 g/kg b.w.	23.5	22	84	89
	3.	7 g/kg b.w.	27.5	26.5	94	109
	4.	7 g/kg b.w.	26.5	25	76	87
	5.	8 g/kg b.w.	31	30	119	115
	6.	8 g/kg b.w.	29	28.5	115	97
	7.	9 g/kg b.w.	33.5	32.5	90	93
	8.	9 g/kg b.w.	33	31.5	95	99

**Table 7:** Daily water consumption (mL/day) in groups of four animals following administration of *Calendula arvensis*, *Polygonum hydropiper*, and *Agrimonia pilosa* tinctures (groups 1, 2, and 3), and physiological saline (group M—control).

Group	Day													
	1	2	3	4	5	6	7	8	9	10	11	12	13	14
1	20	50	20	20	20	20	20	25	25	25	25	20	30	10
2	20	20	20	30	30	20	20	20	25	25	35	20	30	10
3	20	50	20	20	20	25	20	20	30	25	30	25	20	15
M	30	40	10	30	25	30	30	30	35	30	30	25	30	15

**Table 8:** Food consumption (g/day) across groups of four animals following administration of tinctures of *Calendula arvensis*, *Polygonum hydropiper*, and *Agrimonia pilosa* (groups 1–3) and physiological saline (group M—control).

Group	Day													
	1	2	3	4	5	6	7	8	9	10	11	12	13	14
1	19	18	16	18	20	22	22	22	24	26.5	20	21	21	21
2	10	17	20	22	25	23	23	24	23	22	22	25	24	24.5
3	14	15	15	19	19	21	20	21	21	22	23	21	19	20
M	20	20	20	22	22	21	22.5	27	25	22	24	21	22	21.5

**Table 9:** Mean body weight (g) recorded daily in the experimental groups (*Calendula arvensis*, *Polygonum hydropiper*, *Agrimonia pilosa*).

Day	*Calendula arvensis*, Mean ± SD	*Polygonum hydropiper*, Mean ± SD	*Agrimonia pilosa*, Mean ± SD
D1	31.000 ± 3.1623	31.250 ± 0.5000	32.000 ± 2.1602
D2	31.125 ± 3.0653	30.125 ± 0.6292	31.250 ± 1.7078
D3	31.250 ± 2.9861	30.250 ± 0.5000	30.875 ± 2.2500
D4	31.375 ± 3.1983	29.250 ± 1.2583	31.000 ± 2.1602
D5	30.750 ± 2.2174	29.250 ± 1.2583	31.125 ± 2.1360
D6	30.250 ± 2.2174	28.750 ± 1.2583	30.500 ± 1.3540
D7	30.125 ± 2.2127	28.375 ± 1.4930	30.625 ± 1.4930
D8	30.625 ± 2.0156	28.625 ± 2.2867	31.125 ± 0.8539
D9	30.625 ± 2.1747	28.750 ± 1.8484	31.000 ± 1.3540
D10	31.000 ± 2.5820	28.000 ± 2.4495	31.250 ± 0.8660
D11	31.375 ± 2.2867	28.625 ± 2.2867	31.250 ± 1.1902
D12	31.875 ± 2.9545	28.125 ± 2.6575	31.625 ± 1.1087
D13	31.250 ± 2.3979	29.000 ± 2.5820	30.875 ± 1.9311
D14	31.250 ± 2.3629	29.500 ± 1.7321	29.875 ± 2.9545

**Table 10:** Common clinical signs recorded during observation.

Clinical Observation	Observed Signs	Involved System(s)
Respiratory	Dyspnea (abdominal breathing, gasping), apnea, cyanosis, tachypnea, nasal discharge	CNS, pulmonary, cardiovascular
Motor activity	Decreased/increased activity, somnolence, loss of righting reflex, anesthesia, catalepsy, ataxia, abnormal locomotion, prostration, tremors, fasciculations	CNS, somatomotor, sensory, neuromuscular, autonomic
Convulsions	Clonic, tonic, tonic–clonic seizures, asphyxial seizures, opisthotonus	CNS, neuromuscular, autonomic, respiratory
Reflexes	Corneal, righting, myotatic, light reflex, startle reflex	CNS, sensory, autonomic, neuromuscular
Ocular signs	Lacrimation, miosis, mydriasis, exophthalmos, ptosis, opacity, iritis, conjunctivitis, chromodacryorrhea, photophobia	Autonomic, irritative
Cardiovascular signs	Bradycardia, tachycardia, arrhythmia, vasodilation, vasoconstriction	CNS, autonomic, cardiovascular, pulmonary
Salivation	Excessive salivation	Autonomic
Piloerection	Rough/dry hair	Autonomic
Analgesia	Decreased response to stimuli	CNS, sensory
Muscle tone	Hypotonia, hypertonia	Autonomic
Gastrointestinal and renal	Soft stools, diarrhea, emesis, diuresis, rhinorrhea	CNS, autonomic, sensory, gastrointestinal motility, renal
Skin	Edema, erythema	Tissue damage, irritation

## Data Availability

To promote transparency and reproducibility, we will provide a detailed data availability statement. The files and data are in the physical and electronic archive of the University of Medicine and Pharmacy Craiova and can be requested from the corresponding author. All data generated or analyzed during this study are included in this article. Further inquiries can be directed to the corresponding author.
